# Chinese Sumac (*Rhus chinensis* Mill.) Fruits Prevent Hyperuricemia and Uric Acid Nephropathy in Mice Fed a High-Purine Yeast Diet

**DOI:** 10.3390/nu16020184

**Published:** 2024-01-05

**Authors:** Nan Ma, Shengbao Cai, Yilin Sun, Chuanqi Chu

**Affiliations:** Faculty of Food Science and Engineering, Kunming University of Science and Technology, Kunming 650500, China; mn1998spyj@163.com (N.M.); caikmust2013@kmust.edu.cn (S.C.);

**Keywords:** *Rhus chinensis* Mill., hyperuricemia, uric acid nephropathy, xanthine oxidase

## Abstract

Hyperuricemia (HUA) is a prevalent chronic disease, characterized by excessive blood uric acid levels, that poses a significant health risk. In this study, the preventive effects and potential mechanisms of ethanol extracts from Chinese sumac (*Rhus chinensis* Mill.) fruits on HUA and uric acid nephropathy were comprehensively investigated. The results demonstrated a significant reduction in uric acid levels in hyperuricemia mice after treatment with Chinese sumac fruit extract, especially in the high-dose group, where the blood uric acid level decreased by 39.56%. Visual diagrams of the kidneys and hematoxylin and eosin (H&E)-stained sections showed the extract’s effectiveness in protecting against kidney damage caused by excessive uric acid. Further investigation into its mechanism revealed that the extract prevents and treats hyperuricemia by decreasing uric acid production, enhancing uric acid excretion, and mitigating the oxidative stress and inflammatory reactions induced by excessive uric acid in the kidneys. Specifically, the extract markedly decreased xanthine oxidase (XOD) levels and expression in the liver, elevated the expression of uric acid transporters ABCG2, and lowered the expression of uric acid reabsorption proteins URAT1 and SLC2A9. Simultaneously, it significantly elevated the levels of endogenous antioxidant enzymes (SOD and GSH) while reducing the level of malondialdehyde (MDA). Furthermore, the expression of uric-acid-related proteins NLRP3, ACS, and Caspase-3 and the levels of IL-1β and IL-6 were significantly reduced. The experimental results confirm that Chinese sumac fruit extract can improve HUA and uric acid nephropathy in mice fed a high-purine yeast diet. This finding establishes a theoretical foundation for developing Chinese sumac fruit as a functional food or medicine for preventing and treating HUA.

## 1. Introduction

Hyperuricemia, a chronic disease, occurs when blood uric acid levels exceed the normal range due to disrupted purine metabolism in the body [1]. Patients with hyperuricemia have no obvious symptoms in the early stages, but when uric acid accumulates in the kidneys and joints, it can lead to diseases such as uric acid nephropathy and gouty arthritis, eventually leading to renal failure, joint deformity, and even death, which constitutes a serious public health risk [2,3,4]. According to epidemiological statistics, 10% and 20% of adults in China and the United States suffer from hyperuricemia, respectively [5]. Among these individuals, hyperuricemia significantly elevates the risk of developing conditions like gout, chronic kidney disease, hypertension, cardiovascular disease, and even mortality [6]. Hyperuricemia is mainly caused by excessive uric acid production or reduced uric acid excretion, resulting in elevated blood uric acid levels [7]. Therefore, drugs used clinically to treat hyperuricemia mainly include drugs that promote uric acid excretion such as benzbromarone and xanthine oxidase inhibitors such as allopurinol. However, these drugs can have adverse reactions, such as kidney and liver damage as well as gastrointestinal discomfort [8]. The current research indicates that some natural products have demonstrated effective uric-acid-lowering properties with significant efficacy and minimal side effects [9]. Therefore, the development of natural products with uric-acid-lowering effects is very promising for the treatment of hyperuricemia.

Clinically, hyperuricemia is currently defined as blood uric acid levels exceeding 420 μmol/L in men and 360 μmol/L in women under a normal purine diet [10]. Uric acid (UA) is produced by purine metabolism catalyzed by xanthine oxidase in the liver. Xanthine oxidase (XOD) is a complex yellow protease. It can catalyze xanthine and hypoxanthine to produce uric acid and peroxide free radicals and is an extremely important enzyme in the production of uric acid in the human body [11]. Therefore, the production of uric acid can be effectively reduced by inhibiting xanthine oxidase activity and expression [12]. The kidney is the primary site for uric acid excretion, accounting for 65~75% of the body’s daily excretion [13]. UA excretion and reabsorption are facilitated by various transporters, including ATP-binding cassette super-family G member 2 (ABCG2), uric acid transporter 1 (URAT1), and glucose transporter (SLC2A9) [14]. In a study conducted by Arshad Mehmood and colleagues [15], they observed a significant improvement in hyperuricemia by regulating the expression of uric acid transporter proteins.

Chinese sumac (*Rhus chinensis* Mill.) is a plant of the genus Rhus chinensis in the Anacardiaceae family, extensively cultivated in East Asia and Europe [16]. Its fruits are commonly utilized as natural sources for beverages, spices, and edible fruit oil, and is a valuable economic tree species [17]. It is also recorded in some medical books that its roots, stems, leaves, and fruits have good medicinal value and can prevent hepatitis and jaundice, for example [18,19]. Previous research indicates that Chinese sumac fruit is abundant in polyphenolic flavonoids, with notable biological activities including anti-oxidation and anti-inflammation [20]. These compounds have shown efficacy in mitigating conditions like alcoholic fatty liver, colitis, and gastric issues such as ulcers and liver damage [21,22,23]. However, there have been no studies and reports regarding the uric-acid-lowering effect of Chinese sumac fruit. In our preliminary experiments, we found that Chinese sumac fruit exhibited a significant inhibitory effect on XOD in addition to the mentioned biological activities. Moreover, our prior research demonstrated that Chinese sumac fruit effectively prevents gout induced by sodium urate by modulating the NF-κB inflammatory pathway and the NLRP3 inflammasome [24]. The NLRP3 inflammasome pathway is the main pathway for uric acid to trigger inflammatory responses in the human body. The expression of proteins related to this pathway is related to various complications caused by hyperuricemia [25]. Hence, we speculate that Chinese sumac fruit can improve hyperuricemia, but this still needs to be confirmed. In this study, hyperuricemia and uric acid nephropathy in mice were established by using a high-purine yeast diet combined with potassium oxoacid. The study aimed to assess the efficacy of Chinese sumac fruit ethanol extract in ameliorating hyperuricemia and uric acid nephropathy, exploring its underlying mechanism. The findings of this study confirm the uric-acid-lowering properties of Chinese sumac fruit, offering theoretical support for its potential use as a functional food or medicinal agent in preventing and treating hyperuricemia and uric acid nephropathy.

## 2. Materials and Methods

### 2.1. Materials and Reagents

Potassium oxonate (PO) and allopurinol (AP) were obtained from Sigma-Aldrich (Shanghai, China). Radio immunoprecipitation assay (RIPA) lysis buffer, superoxide dismutase (SOD), malondialdehyde (MDA), glutathione (GSH) kits, and bicinchoninic acid (BCA) protein assay were sourced from Shanghai Beyotime Co., Ltd. (Shanghai, China). ELISA detection kits, including IL-1β, and IL-6, were constructed from Multi Sciences Biotech Company (Hangzhou, China). Blood urea nitrogen (BUN), uric acid (UA), n-Acetyl-β-d-glucosaminidase (NAG), and Creatinine (Cr) assay kit was purchased from Nanjing Jiancheng Bioengineering Institute (Nanjing, China). SLC2A9 (Cat No. 26486-1-AP), URAT1 (Cat No. 14937-1-AP), and XOD (Cat No. 55156-1-AP) were purchased from Proteintech (Rosemont, IL, USA). The ABCG2 (Catalog: AF5177) were sourced from Affinity (Cincinnati, OH, USA). ASC (Catalog: A1170) and NLRP3 (Catalog: A5652) were constructed by ABclonal (Wuhan, China). Caspase-1 (Catalog: GB11383) was provided by Wuhan Service Biotechnology Co., Ltd. (Wuhan, China). All other reagents used in this experiment were of analytical grade.

### 2.2. Preparation and Characterization of Chinese Sumac Fruit Extract

Chinese sumac fruits were purchased from Kunming Plant Classification Biotechnology Co., Ltd., Kunming, China, in 2020. Chinese sumac fruit extract (CE) is obtained by crushing fresh fruits and extracting them with 80% ethanol. For details, please refer to the method of Ma et al. [24]. Phytochemicals in the extracts were analyzed and characterized using ultra-high performance liquid chromatography–mass spectrometry. Please see the Supporting Materials for the specific methods and results (Figure S1 and Table S1).

### 2.3. Animal Treatment

Fifty SPF-grade male Kunming mice weighing 18–22 g were purchased from Hunan SJA Laboratory Animal Co., Ltd. (Changsha, China), certificate number SCXK (Xiang) 2019-0004. All experimental mice were kept under the same conditions with a temperature of 23 ± 2 °C, a humidity of 40% to 75%, a daily light time of 12 h, and access to water and food ad libitum. The diet was sourced from Beijing Keao Xieli Feed Co., Ltd., Beijing, China.

**Figure 1:**
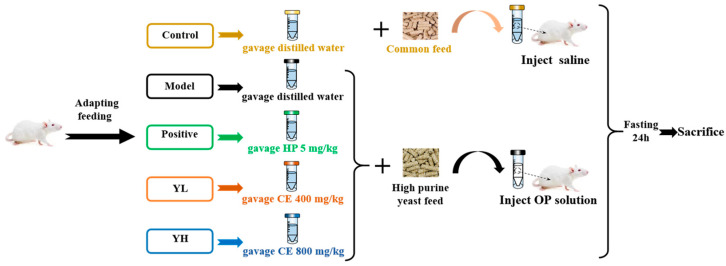
Experimental design and gavage treatment.

The modeling method used in this study refers to the method of Mehmood et al. and was improved according to the previous preliminary experiment [15]. The doses of CE, oxonate potassium, and allopurinol used in this study were determined based on relevant reports in the literature and previous preliminary experimental findings [26,27]. The experimental operation process is shown in Figure 1. Following a week-long adaptation period, 50 mice were randomly divided into five groups, each comprising 10 mice: control group (group C), model group (group M), allopurinol-positive control group (group P), Chinese sumac fruit low-dose group (group YL), and Chinese sumac fruit high-dose group (YH group). After the experiment started, except for group C, the other four groups of experimental mice were fed high-purine yeast feed supplemented with 0.1% adenine and 10% yeast, and drank 5% fructose water. Group C and M were given intragastric normal saline every day, group P was given intragastric allopurinol (AP) solution at a daily intragastric dose of 5 mg/kg. Group YL received daily intragastric administration of fruit extract at a dose of 400 mg/kg, while Group YH was administered CE daily at a dose of 800 mg/kg. After 6 months, except for group C, the other four groups received an intraperitoneal injection of oxonate potassium daily for 7 consecutive days. After 7 days, all mice underwent a 24 h period of fasting without access to food and water. Urine samples were collected during this time. Subsequently, all mice were anesthetized and euthanized by cervical dislocation. Blood was collected, centrifuged, and plasma was obtained for subsequent index measurements. Liver and kidney tissues from the experimental mice were collected, washed with saline, weighed, and the renal index was calculated for each group of mice. The renal index was expressed as a percentage of kidney weight over the whole body weight of the each mouse [28]. A section of the kidney tissues was fixed in 4% paraformaldehyde for pathological analysis, while the remaining tissues were stored at −80 °C for subsequent experiments The experimental protocols were approved by the Animal Experiment Ethics Committee of Kunming University of Science and Technology, and the approval number is PZWH (Dian) K2019-0017). The experimental procedures strictly followed the National Institutes of Health’s Guidelines for The Care and Use of Experimental Animals.

### 2.4. Measurement of Biochemical Indicators

Kits were used to detect the levels of UA, BUN, NAG, Cr, SOD, GSH, MDA, and MDA. IL-1β and IL-6 in the plasma of hyperuricemia mice were detected using ELISA kits (Jiangsu Meimian Industrial Co., Ltd., Yancheng, China).

### 2.5. Renal Histopathological Evaluation

The renal tissue was stained with H&E for histopathological analysis. The kidney tissue was fixed with tissue fixative and embedded in paraffin. The embedded tissue was cut into 5 μm slices and dewaxed for staining. Finally, observations were made using an Olympus IX83 microscope (Tokyo, Japan) under 200× conditions. At the same time, the degree of kidney damage was scored using the method of Sun et al. [28]; 0 is normal (no damage), 1 is 25% (mild), 2 is 25−50% (moderate), 3 is 50−75% (severe), and 4 is more than 75% (very severe).

### 2.6. Western Blot Analysis

The Science Z-II-D cell disruptor was used to disrupt liver and kidney tissue in hyperuricemia mice. The kidney tissue was lysed for 30 min by adding lysis buffer containing protease inhibitors and phosphatase inhibitors. The lysed tissue fluid was centrifuged at 1000× *g* for 5 min at 4 °C. The protein amount of the supernatant was measured using a BCA kit, then the loading buffer was added and boiled for 10 min. After the cooled protein was subjected to SDS-PAGE gel electrophoresis, the target protein on the gel was transferred to a nitrocellulose (NC) membrane (Gelman Laboratory, Ann Arbor, MI, USA) using a membrane transfer instrument, and incubated at 4 degrees Celsius. Incubate with the corresponding primary antibody overnight, and then incubate with the secondary antibody. After adding modified chemiluminescence detection reagent (Marne-La-Vallee, France) to the incubated protein, the VILBER Fusion FX7 imaging system (Millipore, Burlington, MA, USA) was used to analyze the expression of the target protein.

### 2.7. Statistical Analysis

The results of this experiment are expressed as the mean ± standard error (SE). Data analysis was performed using SPSS statistics software (IBM Inc., Armonk, NY, USA) for one-way analysis of variance and Tukey’s multiple comparison test, and values of *p* < 0.05 were considered statistically different. All figures were drawn using OriginLab software (OriginLab, Northampton, MA, USA).

## 3. Results

### 3.1. Effects of CE on the Levels of Plasma and Urinary Uric Acid in Hyperuricemia Mice Fed a High-Purine Yeast Diet

**Figure 2:**
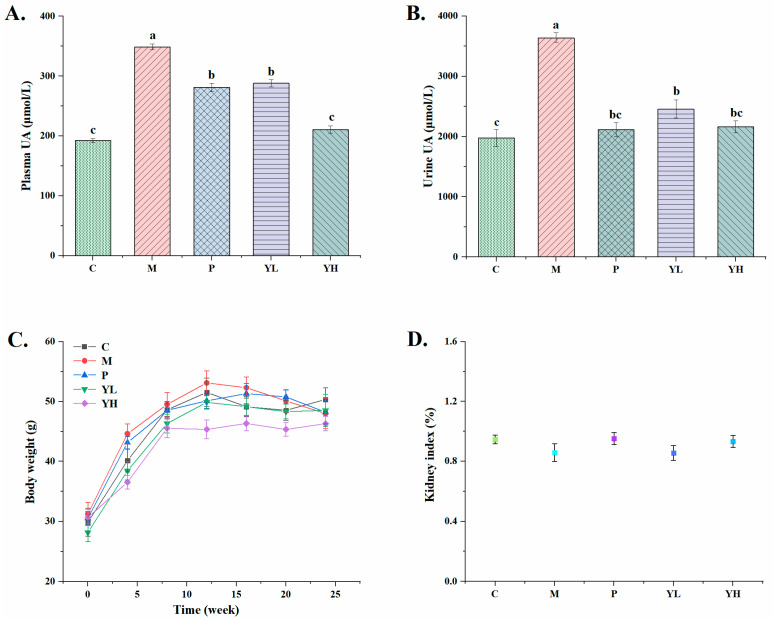
Effects of CE on plasma UA (**A**) and urine UA (**B**) in hyperuricemia mice and the changes in body weight (**C**) and renal index (**D**) of mice in each group during the experiment. All values are expressed as mean ± S.E. (n = 10/group). Different letters on the same bar graph indicate significant differences (*p* < 0.05). C, control group; M, model group; P, positive group (5 mg/kg b.w. of allopurinol). YL, CE low dose group (400 mg/kg b.w. of CE) and YH, CE high dose group (800 mg/kg b.w. of CE).

Figure 2 shows the plasma and urinary uric acid levels of mice with high-purine-yeast-diet-induced hyperuricemia, as well as the body weight changes in each group of mice during the experiment and their renal index. Significant increases were observed in the plasma UA and urine UA levels in group M compared to group C (*p* < 0.05). Pretreatment with allopurinol (group P) and extracts (YL and YH) significantly decreased these indicators (*p* < 0.05). Compared with group P, there was no significant difference in urinary UA levels between groups YL and YH (*p* > 0.05). However, the plasma uric acid level in the YH group was significantly lower than that in the P group (*p* < 0.05). Meanwhile, there were no significant changes in the body weights and kidney indexes of mice in each group during the experimental period. This demonstrated that hyperuricaemia and CE pretreatment had no significant effect on body weight and kidney index.

### 3.2. Effects of CE on Kidney Microstructure in Hyperuricemia Mice Fed a High-Purine Yeast Diet

**Figure 3:**
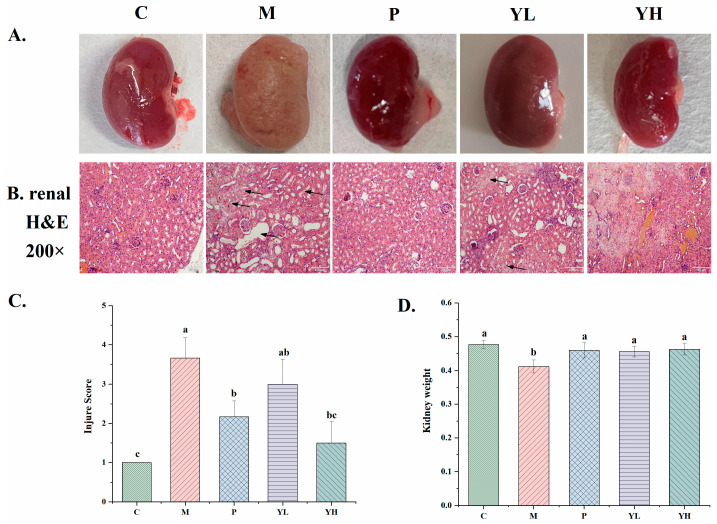
Effect of CE on kidney morphology. (**A**) mouse kidney image; (**B**) kidney H&E staining at 200× magnification; (**C**) histopathological renal injury score; (**D**) kidney weight. Distinct letters indicate significant variations between groups (*p* < 0.05), while identical letters signify no significant differences (*p* > 0.05). C, control group; M, model group; P, positive group (5 mg/kg b.w. of allopurinol). YL, CE low dose group (400 mg/kg b.w. of CE) and YH, CE high dose group (800 mg/kg b.w. of CE).

Figure 3 is a visual map and histopathological section of a mouse kidney after feeding with a high-purine yeast diet. Histopathological sections (Figure 3B) showed that the histological structure of glomeruli, the renal cortex, and medullary tubules in group C appeared normal. In the group M, there were significant changes in the renal tissue structure, including the expansion of renal tubular lesions, tubular degeneration, and tubulointerstitial atrophy. Pre-treatment with allopurinol (group P) and extracts (groups YL and YH) resulted in significant improvements in renal tissue lesions and the overall tissue structure. Notably, the expansion of renal tubular lesions was notably reduced in group P and group YH, with a marked improvement in interstitial atrophy. In particular, group YH showed no evident tissue damage, whereas group YL still exhibited lesions, although these were significantly improved compared to group M. As shown in Figure 3C, the histopathological damage score of kidney tissue in hyperuricemic mice was significantly increased, while, after CE pretreatment, the score was reduced, especially in the high-dose group, which was significantly reduced compared with the M group (*p* < 0.05). This further illustrates the renal protective effect of CE. At the same time, Figure 3D shows the kidney weight of each group. The kidney weight of the hyperuricemic mice in the M group was significantly smaller than that of the other groups, which suggests that kidney atrophy and failure may have occurred.

### 3.3. Effects of CE on Renal Biochemical Indicators in Mice with Hyperuricemia Fed a High-Purine Yeast Diet

**Figure 4:**
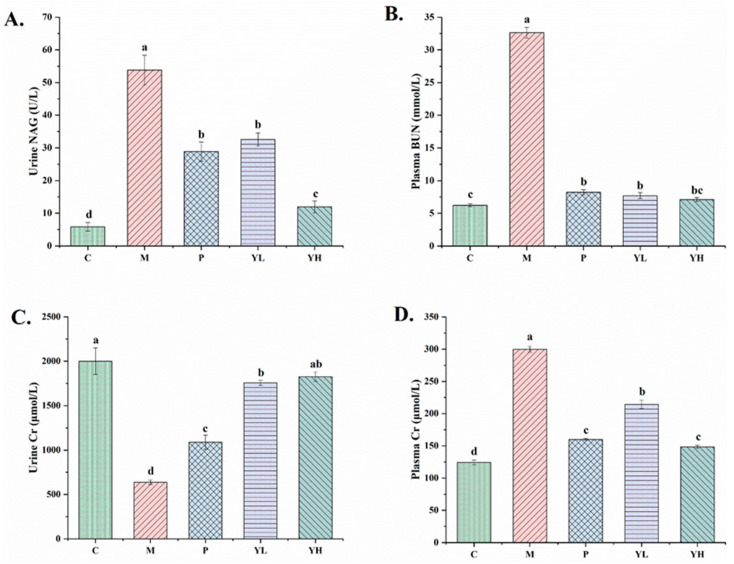
Effects of the CE on urine NAG (**A**) plasma BUN (**B**), urine Cr (**C**) and plasma Cr (**D**) levels in hyperuricemia mice (n = 10/group). Different letters on the same bar graph indicate significant differences (*p* < 0.05). C, control group; M, model group; P, positive group (5 mg/kg b.w. of allopurinol). YL, CE low dose group (400 mg/kg b.w. of CE) and YH, CE high dose group (800 mg/kg b.w. of CE).

Figure 4 shows the results of kidney biochemical indicators in hyperuricemia mice fed a high-purine yeast diet. These indicators include urine NAG, plasma BUN, urine Cr, and plasma Cr levels. In group M, the levels of urine NAG, plasma BUN, urine Cr, and plasma Cr were significantly different from those in group C (*p* < 0.05). Following pretreatment with allopurinol (P group) and extract (YL and YH groups), plasma BUN, Cr, and urine NAG levels were significantly reduced, and urinary Cr levels were significantly increased (*p* < 0.05). Urinary Cr was significantly higher than that in the P group (*p* < 0.05), whereas plasma Cr in the YH group did not significantly differ from the P group (*p* > 0.05). There was no significant difference in plasma BUN in YL and YH groups (*p* > 0.05) compared with P group. Additionally, urinary NAG levels in the YH group were significantly lower than those in the P group (*p* < 0.05).

### 3.4. Effects of CE on the Activity and Expression of XOD in Hyperuricemia Mice Fed a High-Purine Yeast Diet

**Figure 5:**
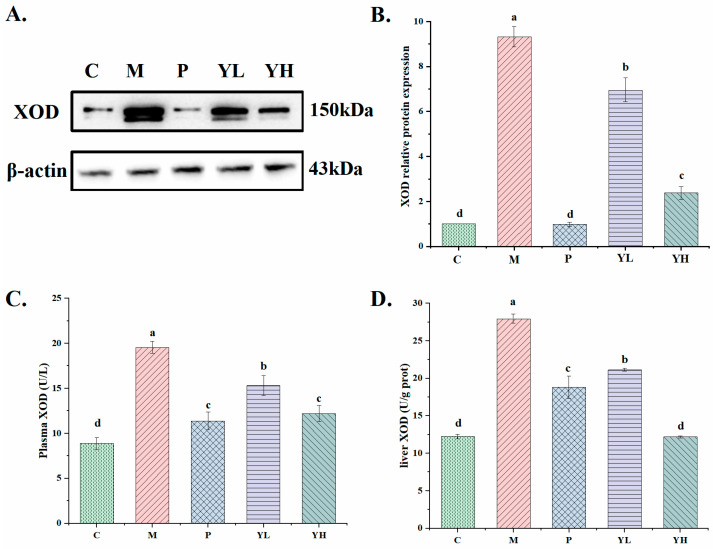
Effects of the CE on XOD expression in the liver tissue (**A**), relative expressions of XOD (**B**), plasma XOD levels (**C**), and liver XOD levels (**D**) in hyperuricemia mice (n = 6/group). The relative expression level was quantified to 1 as the ratio of the blank group to β-actin as a standard for comparison. Different letters on the same bar graph indicate significant differences (*p* < 0.05). C, control group; M, model group; P, positive group (5 mg/kg b.w. of allopurinol). YL, CE low dose group (400 mg/kg b.w. of CE) and YH, CE high dose group (800 mg/kg b.w. of CE).

Figure 5 illustrates the effects of CE on xanthine oxidase (XOD) and expression in the liver and plasma of hyperuricemia mice fed a high-purine yeast diet. Compared with group C, the activity and expression of XOD in the livers and plasma of hyperuricemia mice were significantly increased (*p* < 0.05). Treatment with allopurinol (group P) and the samples (Group YL and YH) significantly reduced these levels (*p* < 0.05). The effects of CE on XOD activity and expression in the liver and plasma were dose-dependent. These values were significantly lower than those in the low-dose group (*p* < 0.05). The XOD activity in the plasma of the high-dose group was not markedly different from that of the allopurinol group (*p* > 0.05). The liver XOD activity was the lowest, comparable to the C group (*p* > 0.05).

### 3.5. Effects of CE on the Expression of Uric Acid Transporter in the Kidney of Mice with Hyperuricemia Induced by a High-Purine Yeast Diet

**Figure 6:**
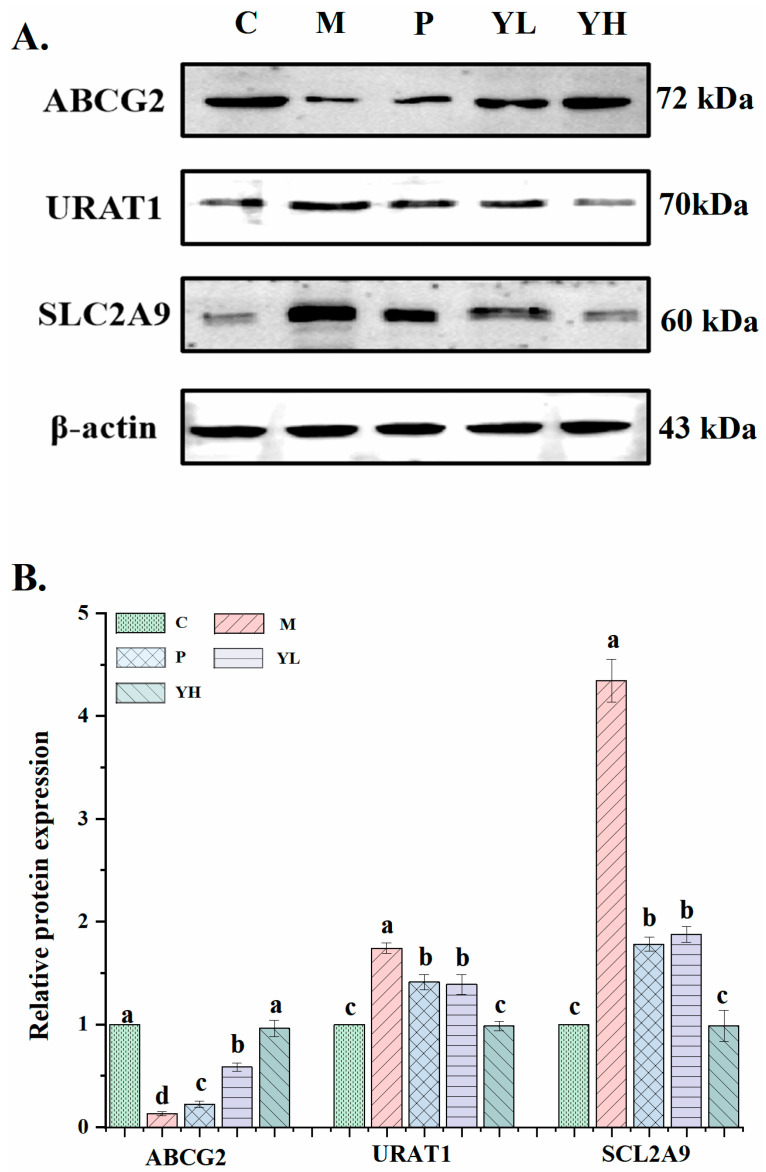
Effect of CE on the expression (**A**) and relative expressions (**B**) of several key proteins related to uric acid transport in the kidney, including ABCG2, URAT1, and SLC2A9 (n = 6/group). The relative expression level was quantified to 1 as the ratio of the blank group to β-actin as a standard for comparison. Different letters on the same bar graph indicate significant differences (*p* < 0.05). C, control group; M, model group; P, positive group (5 mg/kg b.w. of allopurinol). YL, CE low dose group (400 mg/kg b.w. of CE) and YH, CE high dose group (800 mg/kg b.w. of CE).

Figure 6 illustrates the expression of ABCG2, URAT1, and SCL2A9 in the kidney of mice with hyperuricemia. Compared with the corresponding proteins in group C, the expression of ABCG2 in hyperuricemic mice in group M was significantly reduced (*p* < 0.05), and the expression of URAT1 and SLC2A9 was significantly increased. After group P, group YL and group YH were treated with allopurinol and CE, respectively, compared with group M, the expression of ABCG2 was significantly increased, and the expression of URAT1 and SLC2A9 was significantly decreased (*p* < 0.05). Among the P group, YL group, and YH group, the YH group had the highest expression of ABCG (*p* < 0.05) and the lowest expression of URAT1 and SLC2A9 (*p* < 0.05). Moreover, the expression levels of these proteins in the YH group were not significantly different from those in the C group (*p* > 0.05).

### 3.6. Effects of CE on Kidney Oxidative Stress in Hyperuricemia Mice Fed a High-Purine Yeast Diet

**Figure 7:**
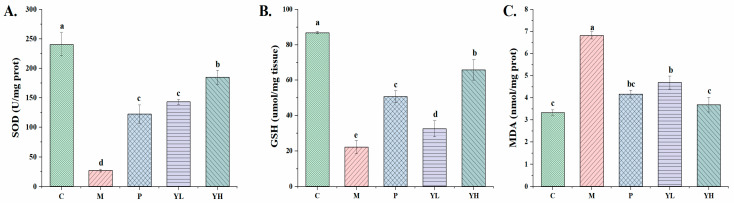
Effects of CE on SOD (**A**), GSH (**B**), and MDA (**C**) levels in the kidney of hyperuricemia mice (n = 10/group). Different letters on the same bar graph indicate significant differences (*p* < 0.05). C, control group; M, model group; P, positive group (5 mg/kg b.w. of allopurinol). YL, CE low dose group (400 mg/kg b.w. of CE) and YH, CE high dose group (800 mg/kg b.w. of CE).

The results of renal oxidative stress indicators (SOD, GSH, MDA) in hyperuricemia mice are shown in Figure 7. In the kidney tissue, compared with group C, the levels of SOD and GSH in the kidney tissue of hyperuricemic mice in group M were significantly reduced (*p* < 0.05), and the level of MDA was significantly increased (*p* < 0.05). However, compared with group M, the levels of SOD and GSH in the kidney tissue of group P and YH were significantly increased and the level of MDA was significantly decreased (*p* < 0.05) after treatment with high purine and high-dose CE, respectively. After low-dose CE treatment, the GSH level of the YL group was significantly increased, the MDA level was significantly decreased, and the SOD level was not significantly different compared with the M group (*p* > 0.05). Moreover, in addition, when comparing the YH group with the P group, except that the MDA content was significantly lower than that of the P group (*p* < 0.05), there was no marked difference in other indicators (*p* > 0.05).

### 3.7. Effects of CE on Inflammatory Factors Levels and NLRP3 Inflammasome-Pathway-Related Proteins Expression in the Kidney of Hyperuricemia Mice Fed a High-Purine Yeast Diet

**Figure 8:**
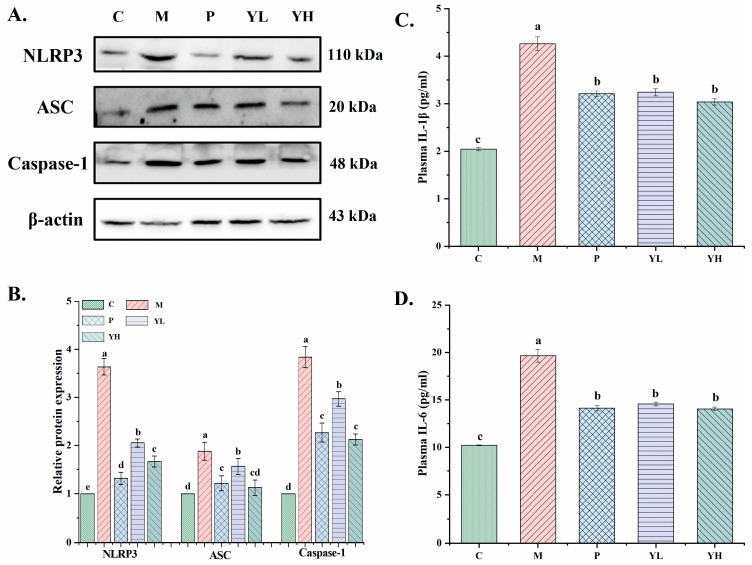
Effects of CE on the expression of NLRP3 inflammasome pathway proteins in kidney tissue (**A**); relative expression levels (**B**). The relative expression level was quantified to 1 as the ratio of the blank group to β-actin as a standard for comparison (n = 6/group). Effects of CE on IL-1β (**C**) and IL-6 (**D**) levels in the plasma of hyperuricemia mice. Different letters on the same bar graph indicate significant differences (*p* < 0.05). C, control group; M, model group; P, positive group (5 mg/kg b.w. of allopurinol). YL, CE low dose group (400 mg/kg b.w. of CE) and YH, CE high dose group (800 mg/kg b.w. of CE).

The expression levels of NLRP3, ASC, and Caspase-3 were detected using Western blot (Figure 8). Compared with group C, the expression levels of NLRP3, ASC, and Caspase-1 in hyperuricemic mice were significantly increased (*p* < 0.05). After AP and CE pretreatment, the expression of these proteins was reduced (*p* < 0.05). Furthermore, the high-dose CE exhibited significantly superior preventive effects compared to the low-dose CE (*p* > 0.05). Compared with the AP group, there was no significant difference in ASC and Caspase-1 expression. Figure 8C,D indicate plasma IL-1β and IL-6 levels in hyperuricemic mice. Plasma IL-1β and IL-6 levels were markedly elevated in group M compared to group C (*p* < 0.05). Conversely, after AP and CE pretreatment, these concentrations were significantly reduced (*p* < 0.05).

## 4. Discussion

The prevalence of hyperuricemia is rising due to overnutrition, primarily caused by excessive purine intake in the daily diet, leading to elevated UA levels [29]. In this study, a prolonged high-purine yeast diet, along with an intraperitoneal injection of the urease inhibitor oxonate potassium and fructose water, was employed to induce hyperuricemia in mice. This model was utilized to assess the anti-uric-acid and nephropathy prevention effects of CE, elucidating its mechanisms of action. This experiment confirmed that CE can effectively improve hyperuricemia and uric acid nephropathy induced by a high-purine yeast diet. The mechanism may be that CE reduces the production of uric acid by reducing the activity and expression of xanthine oxidase in the liver, and regulates the expression of uric-acid-transporter-related proteins, such as URAT1 and ABCG2, that increase renal uric acid excretion and reduce the hyperuricemia caused by a high-purine yeast diet. At the same time, CE reduces uric acid levels by increasing SOD and GSH levels and regulating the NLRP3 inflammasome pathway. Kidney oxidative stress and the inflammatory response caused by elevated uric acid levels protect from the uric acid nephropathy caused by elevated uric acid levels.

Plasma UA level serves as a crucial indicator for assessing hyperuricemia. However, due to species variations, the precise value to define hyperuricemia in mice is not well-defined in mouse models [30]. In this study, the blood UA level in group M was significantly higher than that in group C, approximately five times higher (Figure 3). In comparison with the hyperuricemia model established by Chen et al. [31], the blood UA level of mice in group M in this experiment was higher than that of the hyperuricemic mice. This indicates the hyperuricemia mouse model was successfully established in this experiment. However, CE treatment resulted in lower blood UA levels, confirming the UA-lowering effect of the extract.

The kidney serves as the primary organ for uric acid excretion. More than 70% of uric acid is excreted through the kidneys [32]. Consequently, the kidney plays a pivotal role in maintaining uric acid levels, and enhancing renal excretion stands out as the most crucial method for reducing uric acid levels. Nevertheless, excessively high blood uric acid levels combined with insufficient renal uric acid excretion can result in uric acid deposition within the kidney. This condition can lead to kidney diseases and the onset of uric acid nephropathy [33]. UA nephropathy is a frequent complication of hyperuricemia, causing serious damage to kidney function and potentially leading to severe conditions like kidney stones [34]. Kidneys from the M group appeared white, and it can be seen from the H&E slices that the kidney tissue structure was obviously damaged. This is mainly because, when blood uric acid levels are too high, urate and urate crystals can be deposited in the renal tubular epithelial cells and interstitium, directly causing kidney damage. In the hyperuricemic mice established by Chen et al., a white appearance of the kidneys and obvious damage to the microstructure were also observed [35]. However, the renal tissue damage in the YL and YH groups treated with CE was significantly improved (Figure 3). This demonstrates the efficacy of Chinese sumac fruit in safeguarding kidneys from elevated uric acid damage and preventing hyperuricemia-induced uric acid nephropathy. Chen et al. also achieved similar effects by using Sonneratia apetala seed oil to treat mice with high uric acid levels [35].

To assess and evaluate the effect of Chinese sumac fruit in protecting the kidneys of hyperuricemia mice, we used blood Cr and other related biochemical indicators to evaluate the kidney function. Blood Cr is the metabolite of muscle creatine, and BUN is the end product of protein metabolism. Both are primarily eliminated through glomerular filtration and urine. When the glomerular filtration function is damaged, the Cr and BUN in the blood cannot be excreted out of the body with urine, resulting in a decrease in the level of Cr and BUN in the blood and the Cr level in the urine [36]. Therefore, elevated plasma Cr and urea nitrogen and decreased Cr levels in urine are considered to be the main markers of glomerular filtration impairment [37]. NAG is a highly sensitive indicator of renal tubular function, predominantly found in renal tubular epithelial cells. Increased NAG in urine signifies renal tubular damage [38]. As depicted in Figure 4, the M group exhibited considerable alterations in these indicators compared to the C group, signifying severe kidney-related functional impairment due to hyperuricemia. However, after treatment with the CE, these kidney-related functional indicators were significantly improved, underscoring the extract’s efficacy in preventing the renal function damage induced by hyperuricemia. This result is similar to the results of Mehmood et al., who used stevia residue to treat hyperuricemia mice, and these indicators were significantly improved [15].

Current research shows that, aside from congenital conditions, the two main causes of hyperuricemia are the overproduction of uric acid and underexcretion of uric acid. UA production and excretion are primarily regulated by XOD and renal uric acid excretion [38]. Consequently, current treatments for hyperuricemia commonly involve XOD inhibitors to curb UA production or enhance renal UA excretion for increased UA elimination. Therefore, to delve deeper into the mechanism of Chinese sumac fruit in preventing hyperuricemia and uric acid nephropathy, this study assessed the uric-acid-lowering potential of Chinese sumac fruit ethanol extract through the aforementioned pathways and elucidated its associated mechanisms.

UA is mainly produced in liver tissue [39], with liver XOD levels dozens of times higher than in other tissues. Consequently, inhibiting uric acid production catalyzed by liver xanthine is an important method for reducing uric acid levels. To further investigate the uric-acid-lowering mechanism of CE, we examined liver XOD activity and expression. As depicted in Figure 5, Chinese sumac fruit markedly decreased the expression of XOD in the liver, and significantly inhibited the activity of XOD in plasma and liver. This suggests that inhibiting XOD activity and expression is a key mechanism for reducing uric acid levels in Chinese sumac fruit. Studies have confirmed that polyphenolic compounds have an inhibitory effect on XOD. Our prior research revealed abundant polyphenols in CE. Hence, these phenolic compounds likely contribute to the extract’s XOD inhibition.

UA transporters, including SLC2A9, URAT1, and ABCG2, are vital for renal uric acid excretion [40]. URAT1, an anion exchanger, is responsible for urate reabsorption [41]. Studies have shown that mutations in the URAT1 gene have been linked to hyperuricemia [42]. SLC2A9, another significant uric acid transporter in the kidney, is crucial for uric acid excretion, hyperuricemia treatment, and uric acid reabsorption [43]. ABCG2 is a key protein that regulates the excretion of uric acid. It is called a breast cancer resistance protein and is mainly present in the apical membrane of kidney tissue [44]. In a study of Matsuo et al., it was confirmed that ABCG2 dysfunction can elevate UA levels in the body [45]. To delve deeper into the uric acid excretion mechanism enhanced by Chinese sumac fruit, this experiment assessed the expression of uric acid transporters like URAT1 in hyperuricemia mice the kidneys. In this experiment, after CE treatment, the expression of uric acid transporter proteins, such as ABCG2, in hyperuricemic mice was significantly improved. This confirms that Chinese sumac fruit can effectively prevent and treat hyperuricemia induced by a high-purine yeast diet. It does so by regulating uric acid transporter protein expression and enhancing uric acid excretion. Mehmood et al. also observed similar results in that stevia residue ameliorated hyperuricaemia in mice by upregulating the expression of ABCG2 and downregulating the expression of SLC2A9 and URAT1. [15].

There are also studies showing that UA, as an oxidant, can stimulate the production of ROS and lead to the occurrence of various diseases [46]. Consequently, elevated uric acid levels can result in elevated oxidative stress levels, the depletion of endogenous antioxidant enzymes, ROS generation, and lipid peroxidation. In the present study, we observed that CE pretreatment significantly improved the elevated GSH and SOD levels in the renal tissue of hyperuricemic mice while reducing MDA levels (Figure 7). This confirms that CE can mitigate hyperuricemia and uric acid nephropathy in mice by reducing the level of oxidative stress. Therefore, the regulation of oxidative stress caused by hyperuricemia may be the reason why CE effectively prevents and alleviates hyperuricemia and uric acid nephropathy. Hence, the effective prevention and alleviation of hyperuricemia and uric acid nephropathy by CE may be attributed to its regulation of oxidative stress induced by hyperuricemia. This was also observed in Jiang et al.’s results after treating hyperuricemic mice with gallic acid from Sonneratia apetala leaves [47].

Hyperuricemia can induce renal inflammation by depositing uric acid in the kidney tissue to form sodium urate (MSU) crystals [48]. Macrophages can identify and phagocytose MSU crystals in the renal tubular lumen or interstitial, subsequently leading to NLRP3 inflammasome activation. Once activated, caspase-1 is recruited while promoting IL-1β production [49,50]. Inhibiting the expression of NLRP3 inflammasome-related pathway proteins can effectively alleviate the uric acid nephropathy resulting from elevated uric acid levels and is a feasible strategy in preventing and treating the uric acid nephropathy caused by hyperuricemia [51]. This study revealed a significant upregulation of NLRP3 inflammasome pathway-related proteins in the kidney tissues of hyperuricemia mice. Additionally, serum levels of inflammatory cytokines (IL-1β, IL-6) were notably elevated, indicating severe kidney tissue reactions and confirming the presence of uric acid nephropathy in hyperuricemia mice. Following pretreatment with AP and CE, the expression of NLRP3, ASC, and Caspase-1 proteins and the levels of IL-1β and IL-6 markedly decreased (Figure 8). These findings indicate that CE can decrease the expression of NLRP3 inflammasome pathway-related proteins and reduce inflammatory cytokine levels, leading to improvements in hyperuricemia and uric acid nephropathy. In the study by Chen et al., seed oil also similarly improved kidney damage in mice with high uric acid by regulating NLRP3 inflammasome pathway-related proteins [35].

In summary, the CE inhibits XOD and reduces uric acid production, enhances uric acid excretion, improves kidney tissue oxidative stress levels, regulates the expression of NLRP3-Inflamase-related pathway proteins, and alleviates hyperuricemia induced by a high-purine yeast diet and uric acid nephropathy. This study confirmed that Chinese sumac fruit effectively reduces uric acid levels and ameliorates the uric acid nephropathy induced by high uric acid. While the study established a hyperuricemia model in mice using a high-purine yeast diet to simulate human hyperuricemia, species differences and the presence of urease in mice necessitate further research to determine the specific therapeutic effects and appropriate dosages of the fruit extract for human hyperuricemia [30]. Despite characterizing the types and contents of phenolic substances in the extract, the primary active components responsible for uric acid reduction remain unclear. Further research is necessary to confirm the specific active ingredients in the extract. Furthermore, while the dosage has been validated by toxicology studies, further research is essential to identify the active ingredients in sumac fruit extract and establish safe dosages. The findings of this work broaden the application of CE in hyperuricemia treatment. This information could aid in the development of secure hyperuricemia medications.

## 5. Conclusions

**Figure 9:**
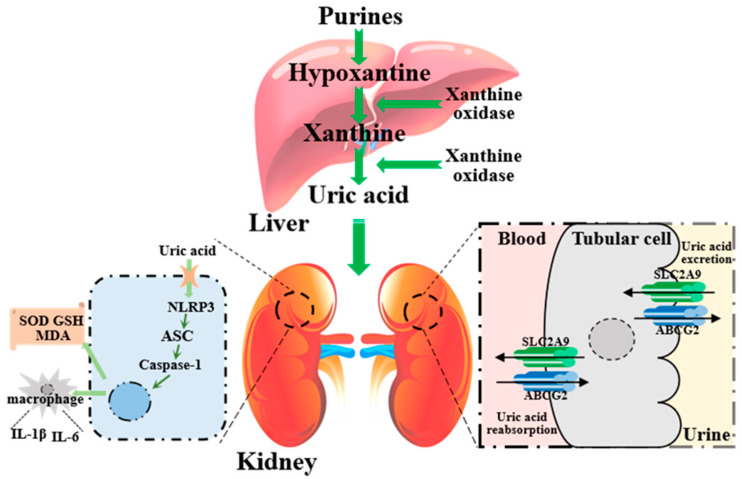
Schematic diagram of the pathogenesis of hyperuricemia and uric acid nephropathy involved in this study.

This study confirmed the effectiveness of CE in effectively ameliorating the hyperuricemia and uric acid nephropathy induced by a high-purine yeast diet. Its effectiveness is primarily achieved by inhibiting XOD, regulating the uric acid transporter protein, enhancing endogenous antioxidant enzymes levels, reducing inflammatory factors, and suppressing NLRP3-inflammasome-related protein expression (Figure 9). This helps to lower body uric acid levels, improving kidney tissue oxidative stress and reducing inflammatory responses. These results provide a basis for further functional research into the use of Chinese sumac.

## Data Availability

The data sets generated and/or analyzed during the current study are either shown in the manuscript and Supplementary Materials or available from the corresponding author on reasonable request.

## Supplementary Materials

The following supporting information can be downloaded at: https://www.mdpi.com/article/10.3390/nu16020184/s1, Figure S1: Chromatogram of ethanol extract from Chinese sumac fruits; Table S1: Phytochemical identification of the ethanol extract from Chinese sumac fruits by UHPLC-ESI-HRMS/MS.
