# Supplementary material for: Chinese Sumac (Rhus chinensis Mill.) Fruits Prevent Hyperuricemia and Uric Acid Nephropathy in Mice Fed a High-Purine Yeast Diet
Source: Nutrients. 2024 Jan 5;16(2):184. doi: 10.3390/nu16020184 (PMC10819650; doi:10.3390/nu16020184)

SUPPORTING INFORMATION

Chinese Sumac (*Rhus chinensis Mill.*) Fruits Prevent Hyperuricemia and Uric Acid Nephropathy in Mice Fed a High-Purine Yeast Diet

Nan Ma, Shengbao Cai, Yilin Sun, Chuanqi Chu*

Faculty of Food Science and Engineering, Kunming University of Science and Technology, Kunming, People's Republic of China, 650500.

* Correspondence：chuanqichu@aliyun.com

Characterization of the major bioactive compounds in the ethanol extract of Chinese sumac fruits

The major bioactive compounds in the ethanol extract of Chinese sumac fruits were firstly separated by using a Thermo Fisher Ultimate 3000 UHPLC System (Thermo Fisher Scientific, Germany) with an Agilent Zorbax SB-C18 column (1.7 μm, 2.1 mm × 100 mm), and then characterized by a Q-Exactive Orbitrap mass spectrometer (Thermo Fisher Scientific, Bremen, Germany) in the negative mode. The HPLC parameters were as follows: mobile phases, 0.1% formic acid in water (A) and acetonitrile (B); flow rate, 0.1 mL/min; elution procedure, 0–2min, 5% B; 2–8 min, 5%–30% B; 8–12 min, 30%–50% B; 12-15min, 50%; 15-18 min, 50%-5%; 18-20 min, 5%; column temperature, 30°C; volume of sample injection, 2.0 μL. Mass parameters were set as follows: full MS scan range, 50–1000 m/z; auxiliary gas flow, 9 L/min; sheath gas flow rate, 33 L/min; sweep gas, 4 L/min; S-lens RF level, 50%; spray voltage, 3.3 kV, capillary temperature, 330 °C; heater temperature, 360 °C.

Fig. S1 Chromatogram of ethanol extract from Chinese sumac fruits. Peak identification and their MS data are shown in Table S1.

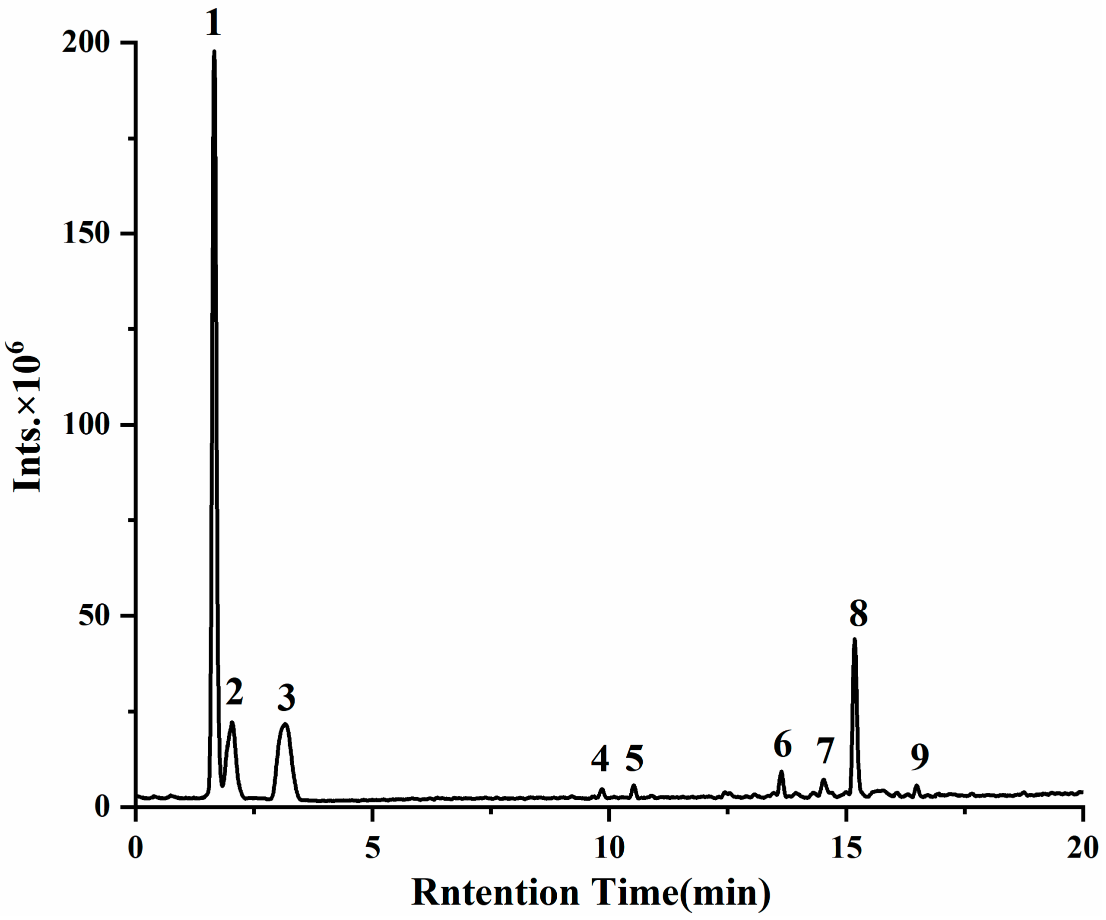

Table S1 Phytochemical identification of the ethanol extract from Chinese sumac fruits by UHPLC-ESI-HRMS/MS

Peak No.	Compounds	RT (min)	Molecular formula	[M−H] − (m/z)	MS/MS ion fragments	mg/g of dry extract	Average percentage (%, Total identified phenolic content)
1	Malic acid	1.66	$C_4H_6O_5$	133.0130	71.0123(100),72.9917(27.45)	---	---
2	Citric acid	2.04	$C_6H_8O_7$	191.0188	57.0332(85.56),67.0175(42.45)	---	---
3	Gallic acid	3.16	$C_7H_6O_5$	169.0132	69.0331(100),124.0153(91.18)	38.21±0.34	47.54
4	Protocatechuic acid	6.78	$C_7H_6O_4$	153.0182	108.0203(100),78.9576(91.18)	4.90±0.16	6.09
5	Digallic acid	9.84	$C_{14}H_{10}O_9$	321.0250	125.0231(100),169.0131(25.58)	4.07±0.10	5.06
6	Trigalloyl glucose	10.51	$C_{27}H_{24}O_{18}$	635.0889	169.0132(100),483.0791(17.62)	4.91±0.14	6.11
7	Myricetin-3-*O*-rhamnoside	13.63	$C_{21}H_{20}O_{12}$	463.0881	316.0221(100),317.0273(30.24)	7.87±0.18	9.80
8	Quercetin-3-O-rhamnoside	15.18	$C_{21}H_{20}O_{11}$	447.0929	300.0272(100),301.0339(59.23)	18.28±0.28	22.75
9	Kaempferol-3-*O*-hexoside	16.49	$C_{21}H_{20}O_{10}$	431.0981	284.0324(100),285.0395(84.51)	2.13±0.20	2.65

RT: retention time; Values are expressed as the mean ± S.D. (*n* = 3, μg/g of dry extract); Gallic acid standard was used for quantifying the compounds 3,5;6; protocatechuic acid standard was used for quantifying the compound 4; myricetin-3-O-rhamnoside standard was used for quantifying the compounds 7; quercetin-3-*O*-rhamnoside standard was used for quantifying the compounds 8; kaempferol standard was used for quantifying the compounds 9.

Raw images of western blot in Figure 5

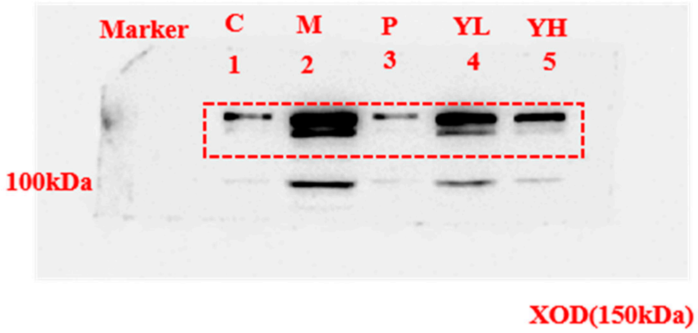

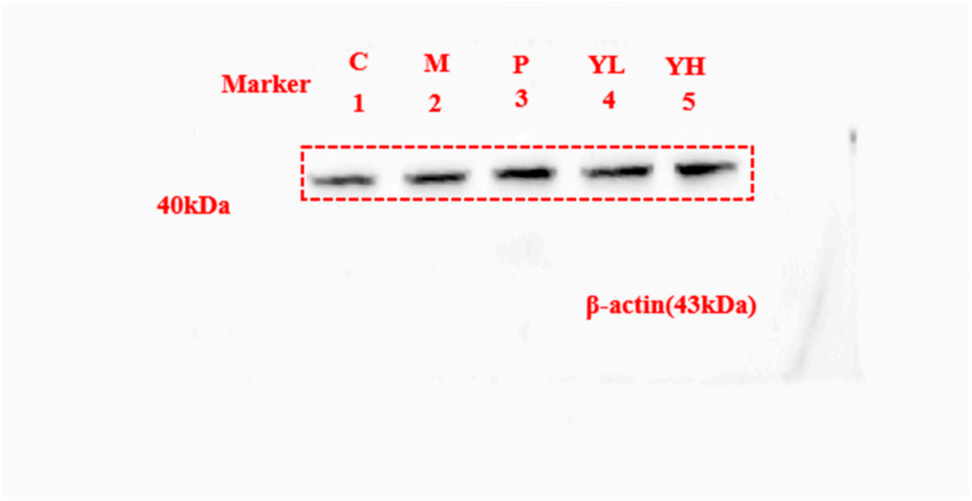

Raw images of western blot in Figure 6

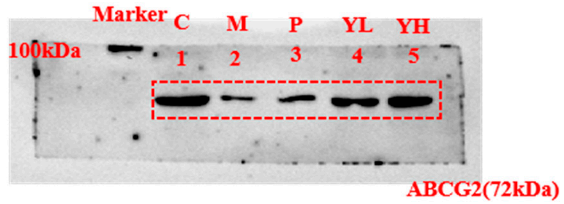

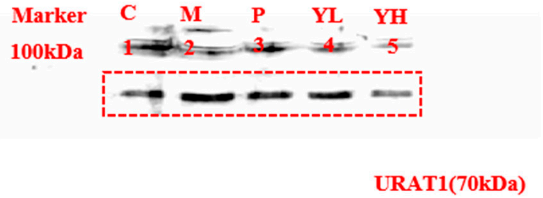

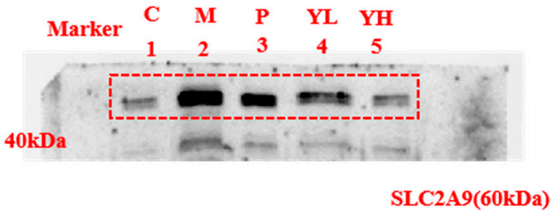

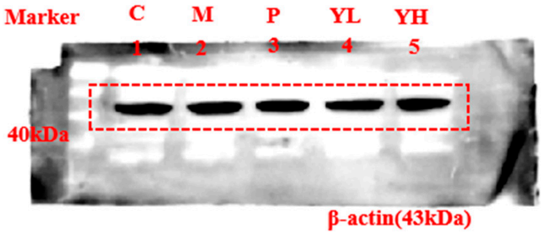

Raw images of western blot in Figure 8

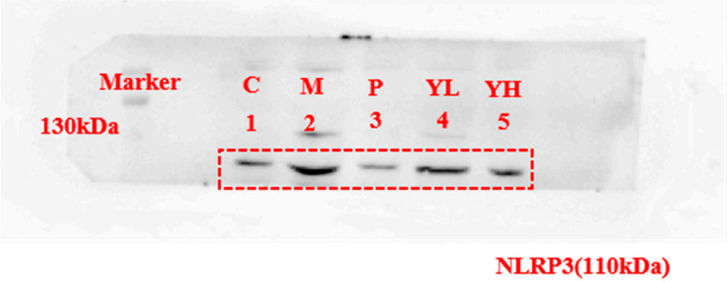

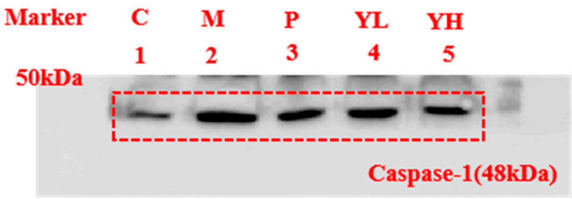

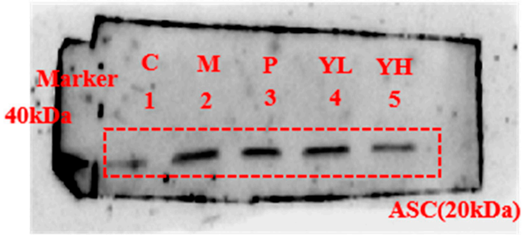

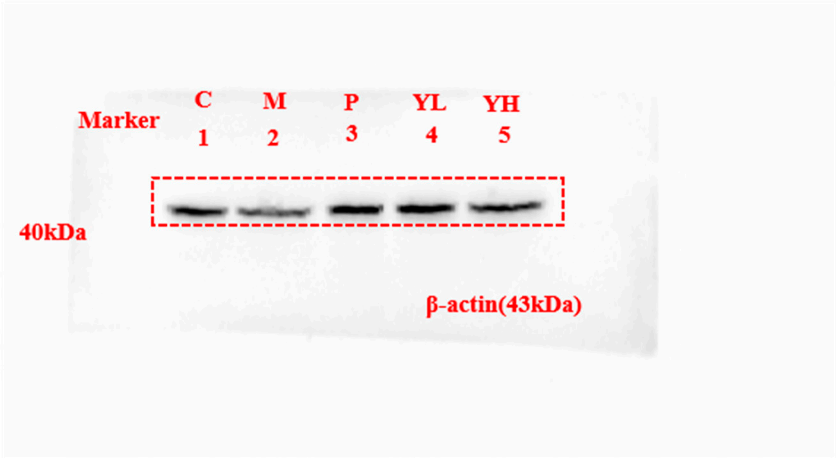

Supplement: Supplementary file 1 [file nutrients-16-00184-s001.zip › nutrients-2733130-supplementary.pdf]
